# Supplementary material for: Contrasting Volatilomes of Livestock Dung Drive Preference of the Dung Beetle Bubas bison (Coleoptera: Scarabaeidae)
Source: Molecules. 2022 Jun 28;27(13):4152. doi: 10.3390/molecules27134152 (PMC9268081; doi:10.3390/molecules27134152)
Supplement: Supplementary file 1 [file molecules-27-04152-s001.zip › molecules-1767461-supplementary.pdf]

Contrasting Volatilomes of Livestock Dung Drive Preference of the Dung Beetle *Bubas bison* (Coleoptera: Scarabaeidae)

Supplementary materials

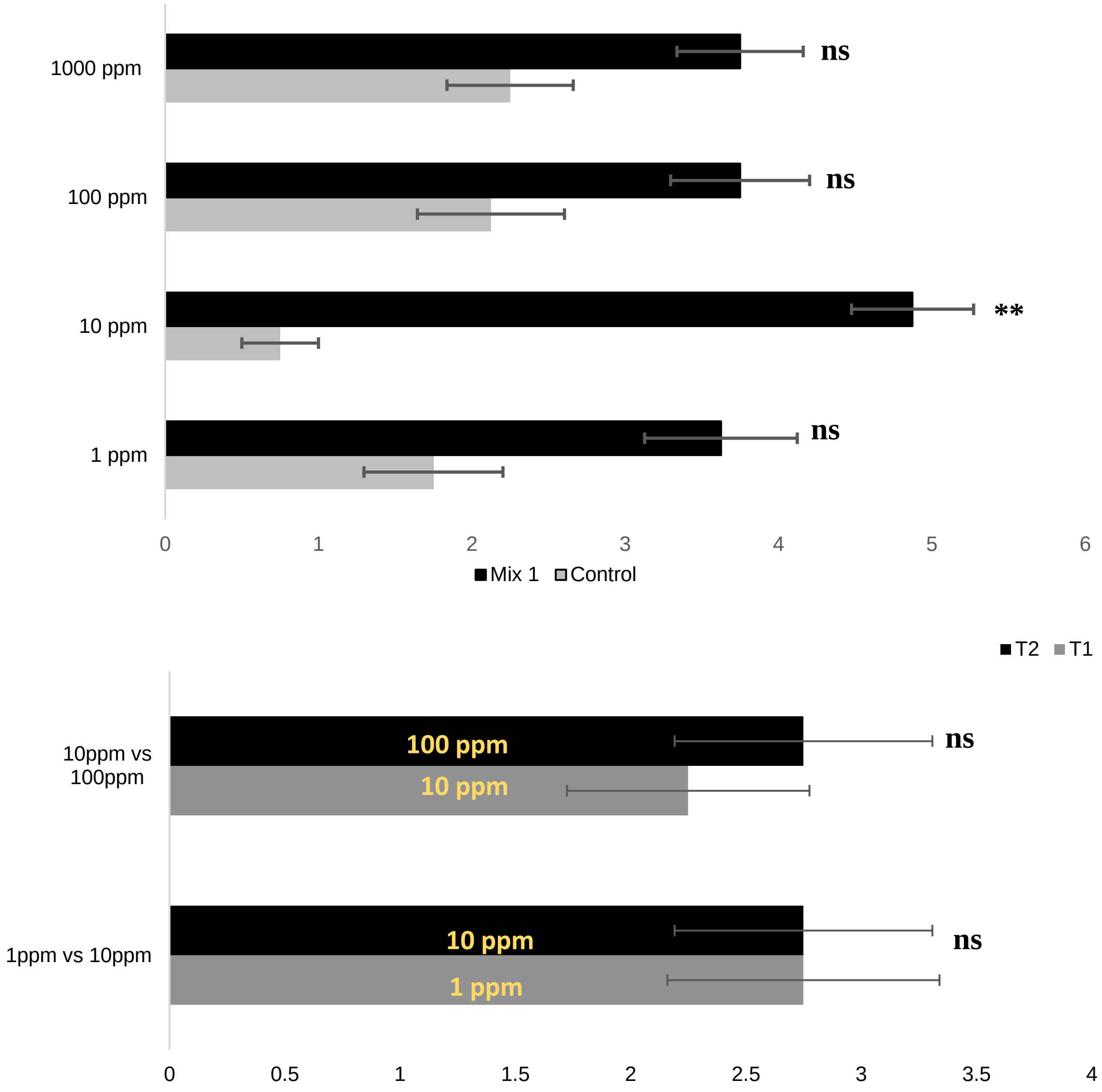

Figure S1. Concentration determination for the VOC assay (**, $p < 0.01$ and 'ns', not significant). Based on this preliminary assay, 10 ppm concentration was selected for the selected volatile compound assay.

Table S1. Features identified across fresh cattle, horse and sheep volatilomes. Compounds verified by standards injections are marked with an asterisk '*'. Compounds that are suspected of being behaviorally active are marked with '#'.

	Compounds	Retention time (± 0.02 min)	Retention index		Dung type		
			Calculated	NIST hit	Cattle	Horse	Sheep
	Alkanes						
1	Heptane	8.11	-		*		*
2	2,3,3-Trimethylpentane	8.239	-				*
3	cis-1-Ethyl-2-Methylcyclopentane	10.45	815	820		*	
4	2,3,5-Trimethylhexane	10.47	816	812			*
5	5,5-Dimethyl-1-vinylbicyclo[2.1.1]hexane	14.88	924	921	*		
6	2,5,6-Trimethyldecane	18.92	1021			*	
7	2,6-Dimethylnonane	19.03	1024	1018	*		*
8	Dodecane	25.97	1200	1200	*	*	*
9	Cyclododecane	31.50	1357	1330		*	
10	2,6,10-Trimethyltridecane	35.00	1463	1449	*	*	
11	n-Decylcyclopentane	36.34	1505	1543			*
12	Octadecane	44.84	1800	1800		*	
	Alkenes						
13	2-Pentene	7.51	-			*	*
14	2-Methyl-2-butene	8.64	-			*	*
15	(E)-4-Octene	9.75	-		*	*	
16	(E)-2-Octene	9.86	801	798	*	*	
17	(Z)-2-Octene	10.12	807	804	*	*	
18	1,6-Dimethylhepta-1,3,5-triene	11.70	846	837	*	*	
19	1,2,4,4-Tetramethylcyclopentene	12.34	862	857		*	*
20	Unidentified monoterpene 1	15.02	927	929	*		*
21	Unidentified monoterpene 2	15.18	931	929	*		*
22	*α*-Pinene*	15.52	939	937	*	*	*
23	(-)-*β*-Citronellene	15.86	947	943	*	*	
24	Camphene	16.18	955	952	*		*
25	2,4(10)-Thujadiene	16.39	960	956	*		
26	cis-3,7-Dimethyl-2-octene	16.81	970	970	*	*	*
27	*β*-Pinene*	17.36	983	979	*		*
28	*β*-Myrcene	17.77	993	991	*		
29	2,6-Dimethyl-2-trans-6-octadiene	18.19	1003	991	*		
30	*α*-Phellandrene*	18.44	1009	1005	*	*	
31	3-Isopropyl-6-methylcyclohexene	18.55	1012	1004		*	
32	(3E)-3-Ethyl-2-methyl-1,3-heptadiene	18.72	1016	-		*	
33	(2E,4E)-3,7-Dimethylocta-2,4-diene	19.16	1027	1025		*	*
34	*p*-Cymene*	19.27	1029	1025	*	*	*

35	Limonene*#	19.46	1034	1030	*	*	*
36	Eucalyptol*#	19.60	1038	1032	*	*	*
37	4-Methyl-1-undecene	20.48	1060	1085	*	*	
38	Fenchol*	22.95	1171	1168	*		
39	Camphor	24.26	1156	1145	*		
40	1-Tridecene	29.30	1293	1292		*	
41	δ-Elemene	31.29	1351	1338		*	
42	*β*-Cubebene	32.69	1392	1386	*		*
43	(3E)-3-Tetradecene	32.72	1393	1385		*	
44	Caparratriene	33.28	1410	1493	*	*	*
45	Caryophyllene	33.79	1426	1419		*	
46	Guaia-6,9-diene	33.89	1429	1443	*	*	
47	Aromandendrene	34.25	1440	1440	*	*	*
48	Alloaromadendrene	34.89	1460	1461	*	*	*
49	Valencene	35.61	1482	1492		*	
50	(Z)-7-Hexadecene	35.96	1493	1566			*
51	δ-Guaiene	36.69	1516	1505	*	*	*
52	D-Germacrene	36.20	1500	1481			*
53	Guaia-3,9-diene	38.58	1591	1556			*
54	Neophytadiene	44.95	1842	1837		*	*
55	3,7,11,15-Tetramethylhexadec-2-ene	46.12	1849	1430		*	*
	Alkynes						
56	3-Nonyne	15.26	933	915		*	
57	1-Pentadecyne	38.58	1579	1518		*	*
58	9-Octadecyne	41.80	1689			*	
59	3-Octadecyne	45.94	1842		*		
	Alcohols						
60	2-Methyl-2-propanol	3.58	-				*
61	1-Pentanol	7.64	-		*	*	*
62	2,4-Dimethyl-1-heptanol	18.08	1000		*		*
63	3,4-Dimethylcyclohexanol	22.73	1116	1126	*	*	*
64	Phenylethyl Alcohol	22.83	1119	1116		*	*
65	Borneol (terpene alcohol)	25.03	1176	1166	*		
66	Cubenol	50.57	1647	1640			*
67	n-Pentadecanol	44.57	1790	1778			*
	Aldehydes						
68	Benzeneacetaldehyde	20.05	1049	1045			*
69	2-Isopropenyl-5-methylhex-4-enal	21.33	1081	-			*
70	3-Ethylbenzaldehyde	24.84	1171	1168	*	*	*
71	*β*-Cyclocitral	27.07	1231	1220	*	*	*
72	2,6,6-Trimethyl-1-cyclohexene-1-acetaldehyde	28.44	1265	1254	*	*	*
	Ketones						
73	2-Butanone*#	4.13	-		*	*	*
74	Ethyl ketone	6.38	-			*	
75	3-Pentanone	6.40	-				*

76	2-Methyl-4-octanone/5-Nonanone	7.72	-			*	*
77	3-Methyl-2-hexanone	8.18	-			*	
78	2-Hexanone	9.48	-				*
79	2,2,4,7-Tetramethyl-3,5-octanedione	11.28	836	-		*	
80	1-(2-methylcyclopenten-1-yl) ethanone	12.18	858		*		
81	5-Ethyl-2-octen-4-one	12.60	868	-		*	*
82	2-Heptanone	13.52	891	891		*	*
83	2-Methyl-6-heptanone	16.23	956	956		*	*
84	6-Methyl-5-heptene-2-one	17.56	988	986	*		*
85	2-Octanone	17.74	992	990		*	*
86	1,1,3-Trimethyl-2-cyclohexanone	19.70	1040	1036			*
87	*β*-Isophorone	19.91	1045	1044			*
88	2-Nonanone	21.84	1093	1092		*	*
89	3-Thujen-2-one	24.92	1173	1171	*		
90	Benzylacetone	27.81	1251	1232			*
91	Dihydro-β-ionone	34.59	1451	1433	*	*	*
92	*β*-Ionone	36.12	1498	1491		*	*
93	5,6,7,7a-tetrahydro-4,4,7a-trimethyl-2(4H)-benzofuranone	37.82	1554	1538		*	
	Aromatic hydrocarbons						
94	Toluene*#	8.73	-		*	*	*
95	*p*-Xylene	12.43	864	865	*	*	*
96	*m*-Xylene	12.76	872	866	*	*	*
97	Styrene	13.66	894	893	*	*	*
98	*m*-ethyl-toluene	16.63	966	957	*		
99	Mesitylene	16.92	972	972			*
100	*p*-Cymenene	21.75	1091	1090	*	*	*
101	Naphthalene	25.79	1196	1182	*		
102	δ-Cadinene	37.36	1533	1524			*
	Carboxylic acids						
103	Acetic acid	4.62	-			*	*
104	2-Oxo-4-phenyl-3-butenoic acid	29.29	1292		*		
	Esters						
105	Methyl 2,2-dimethylbutanoate	9.06	-				*
106	Propyl propanoate	10.26	810	807			*
107	Ethyl 2-methyl-butanoate	11.88	850	849			*
108	3-Methylbutyric acid ethyl ester	12.01	854	854	*	*	*
109	3-Methylbutyl acetate	12.96	877	866			*
110	Propyl 2-methylbutanoate	15.86	947	946			*
111	Propyl 3-methylbutanoate	15.99	950	946			*
112	3-Methylbutyl 2-methylbutanoate	22.17	1101	1101			*

113	Methyl 3,7,11-trimethyldodecanoate	26.98	1227	-		*	
	Phenols						
114	Phenol*#	17.22	980	980	*	*	*
115	*p*-Cresol*#	21.05 ± 0.07	1075	1077	*	*	*
116	4-Ethylphenol	24.69	1167	1169	*		
	'S' containing compounds						
117	Dimethyl sulfide*	3.20	-				*
118	Dimethyl disulfide*	7.96	-			*	*
119	2-Methylthiophene	8.92	-		*		*
120	3-Methylthiophene	9.19	-				*
121	Allyl Isothiocyanate	13.30	886	886	*	*	*
	Others						
122	3-Methylfuran	4.26	-		*	*	*
123	3-Methylpyrazole	4.44	-				*
124	Methoxy-phenyl- oxime	13.84	898	-	*	*	*
125	*o*-Isopropylanisole	22.57	1112	1118	*	*	
126	Indole*#	29.64	1302	1295		*	*
127	Skatole*#	32.89	1398	1391	*	*	*

Table S2. Features identified across volatilomes of fresh horse dung collected from lucerne hay and pasture feeding animals. Compounds verified by standards injections are marked with an asterisk '*'. Compounds that are suspected of being behaviorally active are marked with '#'.

	Compounds	Retention time (± 0.02 min)	Retention index		Horse diet type	
			Calculated	NIST hit	Lucerne hay	Pasture
	Alkanes					
1	1-Methyl-trans-3-ethylcyclopentane	10.33	801	794	*	
2	1-Methyl-3-ethylcyclopentane	10.67	809	791		*
3	1,1,2,3-Tetramethylcyclohexane	16.52	958	958		*
4	4-Methylnonane	16.76	964	961	*	
5	4,5-Dimethylnonane	20.48	1056	1041	*	
6	4-Methyldecane	20.69	1061	1060	*	
7	2-Methyldecane	20.99	1068	1064	*	
8	2,4-Diisopropyl-1,1-dimethylcyclohexane	21.23	1074	-	*	
9	2,6-Dimethylundecane	21.77	1088	1210	*	
10	4-Ethyldecane	23.73	1140	1141	*	
11	2,3,7-Trimethyldecane	35.28	1467	1466	*	*
	Alkenes					
12	Cyclopentene	3.19	-		*	
13	2-Pentene	7.71	-		*	
14	(E)-4-Octene	9.98	-			*

15	(E)-2-Octene	10.08	-			*
16	(Z)-2-Octene	10.35	801	804	*	*
17	1,2,4,4-Tetramethyl-1-cyclopentene	12.58	859	854	*	
18	*α*-Pinene	15.77	940	937	*	*
19	(-)-β-Citronellene	16.11	948	943	*	*
20	Camphene	16.36	954	952	*	
21	cis-3,7-Dimethyl-2-octene	17.08	972	970	*	*
22	*β*-Pinene	17.63	985	979	*	
23	2,6-Dimethyl-2-trans-6-octadiene	17.88	991	991	*	*
24	*p*-Menth-2-ene	18.44	1005	1004		*
25	3-Carene	18.56	1008	1011	*	
26	*α*-Phellandrene	18.71	1012	1005		*
27	(2E,4E)-3,7-Dimethyl-2,4-octadiene	18.83	1015	1025	*	
28	*p*-Cymene	19.54	1032	1025	*	*
29	Limonene	19.72	1037	1031		*
30	Eucalyptol	19.87	1041	1032		*
31	(Z)-3-Undecene	22.07	1095	1086		*
32	*p*-Mentha-1,4(8)-diene	22.15	1097	1088	*	
33	(3Z,5E)-1,3,5-Undecatriene	25.36	1181	1182	*	
34	3,4-Dimethylcumene	26.41	1209	-	*	
35	1,5,6,7-Tetramethylbicyclo[3.2.0]hepta-2,6-diene	29.55	1296	1280	*	
36	(5E)-5-Tridecene	29.58	1296	1280		*
37	Ylangene	32.19	1373	1372	*	
38	*β*-Cubebene	32.74	1389	1389	*	
39	Bicyclo[10.1.0]tridec-1-ene	33.37	1408	-	*	*
40	β-Gurjunene	34.09	1430	1432	*	
41	Aromadendrene	34.56	1445	1440	*	
42	Patchoulene	35.19	1464	1467		*
43	Chamigrene	35.66	1479	1476	*	
44	α-Selinene	36.15	1493	1494	*	
45	δ-Guaiene	36.60	1509	1505	*	
46	(3E)-3-Octadecene	44.86	1794	1785	*	*
	Alkynes					
47	3-Nonyne	15.51	933	915	*	*
	Alcohols					
48	3,3,6-Trimethyl-1,5-heptadien-4-ol	20.73	1062	1084		*
49	Phenylethyl Alcohol	23.12	1112	1116		*
50	2-Butyl-1-octanol	28.80	1275	1277	*	
51	2-Hexyldecanol	36.46	1504	1504	*	
	Aldehydes					
52	β-Cyclocitral	27.36	1220	1235		*
	Ketones					
53	2-Heptanone	13.79	891	891		*
	Aromatic hydrocarbons					
54	Toluene	8.95	-		*	*
55	*p*-Xylene	13.01	870	865	*	*

56	Styrene	13.91	894	893	*	*
57	1-Ethylidene-1H-indene	30.14	1313	1315	*	
58	1,1,3a-Trimethyl-7-methylenedecahydro-1H-cyclopropa[a]naphthalene	34.20	1434	1435	*	
59	ε-Muurolene	34.83	1453	1459	*	
60	(-)-Aristolene	34.99	1458	1453	*	
61	β-Cadinene	36.71	1512	1518		
62	α-Cadinene	36.97	1521	1538	*	
63	δ-Cadinene	37.67	1544	1524	*	
	Phenols					
64	Phenol	17.47	980	980		*
65	*o*-Cresol	20.54	1057	1054		*
66	*p*-Cresol	21.33	1077	1077		*
67	*p*-Ethylphenol	24.93	1168	1169		*
	'S' containing compounds					
68	Dimethyl sulfide	3.31	-			*
69	Dimethyl disulfide	8.17	-			*
70	3-Ethylthiophene	12.85	866	869		*
71	Dimethyl trisulfide	17.34	978	970		*
	Others					
72	3-Methylpyrazole	4.54	-		*	
73	Benzene	5.68	-		*	
74	2,4-Dimethylfuran	7.18	-		*	
75	Methoxy-phenyl-oxime	14.03 ± 0.04	897		*	*
76	2-Ethylpyridine	14.43	907	906	*	
77	*o*-Decylhydroxylamine	21.55	1082	1100	*	
78	Indole	29.92	1306	1295		*
79	Skatole	33.19	1402	1391		*

Table S3. Relative abundance of the compounds identified in pasture-fed and lucerne hay-fed horse dung via MZmine based feature list.

Compounds	Relative abundance (%)	
	Pasture-fed horse dung	Lucerne-fed horse dung
Dimethyl disulfide	3.27	0
Toluene	59.41	8.33
(Z)-4-Octene	0.35	0.3
(E)-2-Octene	0.12	0
2-Heptanone	1.11	0
Styrene	0.12	0
Methoxy-phenyl-oxime	19.79	4.51
3-Nonyne	9.75	9.03
α-Pinene	2.54	80.39
(-)-β-Citronellene	21	10.92
Camphene	0	1.51
4-Methylnonane	0	2.94
2-Methyl-2-nonene	0.78	5.44
Dimethyl trisulfide	0.33	0
Phenol	42.56	2
β-Pinene	0.58	4.12
2,6-Dimethyl-2-trans-6-octadiene	3.83	0
p-Menth-2-ene	0.69	0.09
α-Phellandrene	1.33	0
p-Cymene	1.1	0.71
Limonene	0.27	0.96
Eucalyptol	1.44	0.84
o-Cresol	0.12	0
4-Methyldecane	0	2.45
2-Methyldecane	0	4.25
p-Cresol	100	0.61
O-Decylhydroxylamine	0	6.66
p-Mentha-1,4(8)-diene	0.13	0.76
p-Ethylphenol	0.74	0
(3Z,5E)-1,3,5-Undecatriene	0	0.72
3,4-Dimethylcumene	0	1.21
Indole	7.67	0
Ylangene	0	0.73
β-Cubebene	0	2.18
Skatole	39.44	0
Bicyclo[10.1.0]tridec-1-ene	0.37	4.17
β-Gurjunene	0	1.82
Aromandendrene	0	100
(-)-Aristolene	0	1.03
2,3,7-Trimethyldecane	0.4	3.61
Chamigrene	0	22.28

2-Hexyldecanol	0	0.22
δ-Cadinene	0	1.07
(3E)-3-Octadecene	1.18	0.11

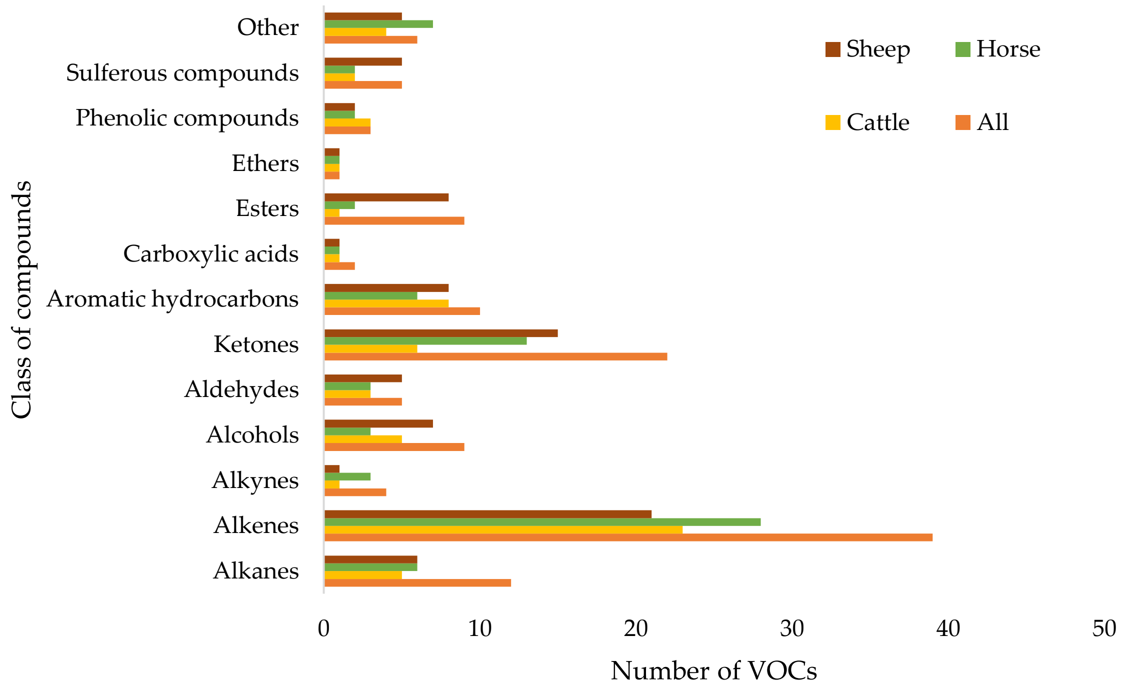

Figure S2. Class of VOCs detected in pasture fed horse, sheep and cattle dung.

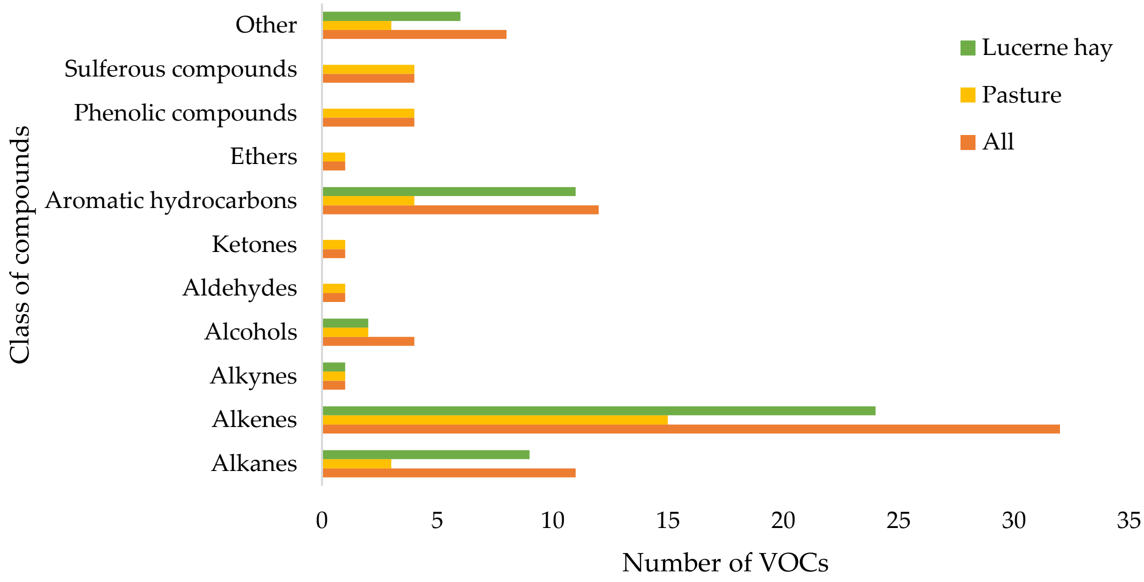

Figure S3. Classes of VOCs detected in pasture- and lucerne hay-fed horse dung.

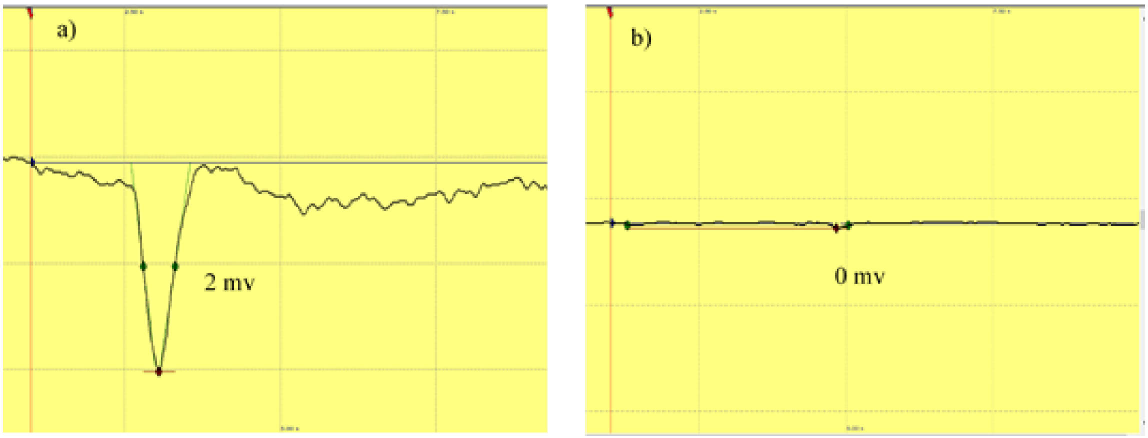

Figure S4. Representative EAG responses of female *B. bison* antennae to a) six-compound mix (10 ppm) and b) control. The stimulus was applied as a puff for 0.2 s using the SYNTEC stimulus controller. (n=7 beetles).

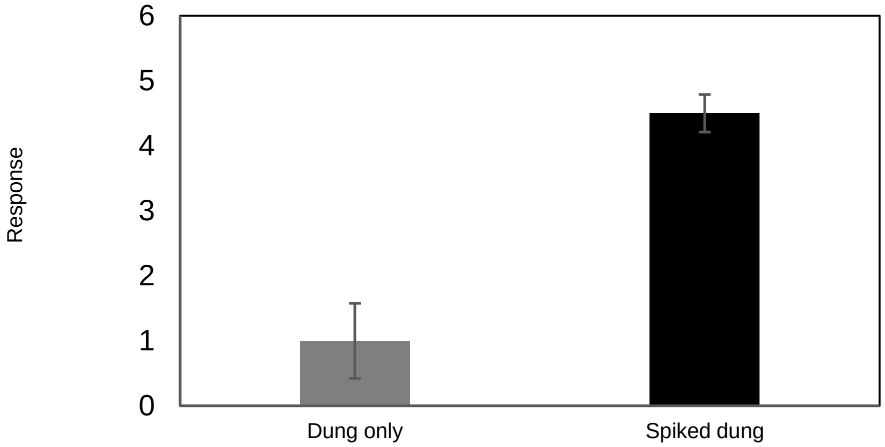

Figure S5. Beetle response to the spiked horse dung assay. Here, dung from horses fed lucerne hay was spiked with toluene, *p*- cresol, phenol and skatole at 10 ppm concentration.
